# Supplementary material for: Detecting misfolded non-covalent lasso entanglements in protein structures, simulation trajectories, and mass spectrometry data
Source: bioRxiv. 2026 Apr 17:2026.04.15.718775. Preprint. [Version 1] doi: 10.64898/2026.04.15.718775 (PMC13105006; doi:10.64898/2026.04.15.718775)
Supplement: Supplement 1 [file media-1.pdf]

Detecting misfolded non-covalent lasso entanglements in protein structures, simulation trajectories, and mass spectrometry data

Ian Sitarik[1,2], Yang Jiang[3,*], Hyebin Song[4], Edward P. O'Brien[1,2,3,5,*]

[1] Institute for Computational and Data Sciences, Pennsylvania State University, University Park, Pennsylvania, United States
[2] National Science Foundation National Synthesis Center for the Emergence of Molecular and Cellular Sciences, Pennsylvania State University, University Park, Pennsylvania, United States
[3] Department of Chemistry, Pennsylvania State University, University Park, Pennsylvania, United States
[4] Department of Statistics, Pennsylvania State University, University Park, Pennsylvania, United States
[5] Bioinformatics and Genomics Graduate Program, The Huck Institutes of the Life Sciences, Pennsylvania State University, University Park, Pennsylvania, United States
* To whom correspondence should be addressed: yuj179@psu.edu, epo2@psu.edu

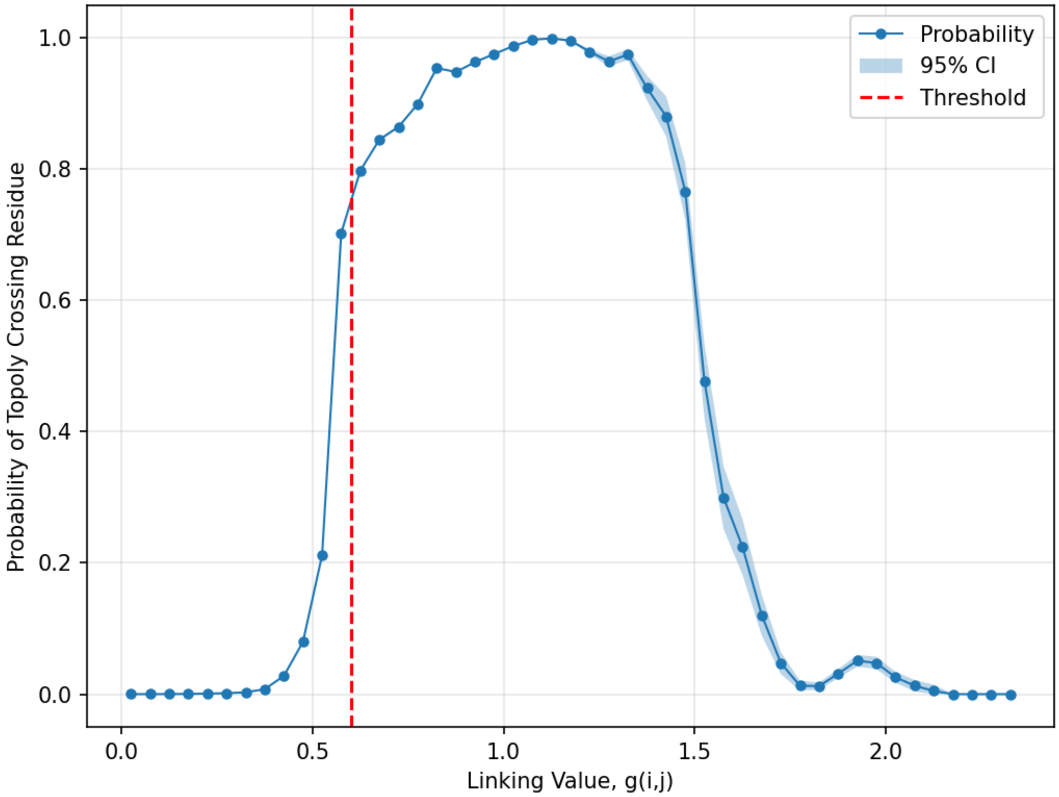

Supplementary Figure 1: The probability of the Topoly python package finding a crossing residue as a function of the absolute value of the Gauss linking value across the last 10 ns of all 1000 trajectories of ePGK. The Topoly default value for the min distance between two crossings was used (10 residues), but we use our terminal and loop buffers of 5 and 4 residues respectfully. Shaded regions are 95% confidence intervals. Red dashed line is the 0.6 threshold for determining the presence of a NCLE. At high values of $|g(i,j)|$ the default min distance between crossings of 10 residues dramatically decreases the accuracy. This parameter is variable in our EntDetect package and we suggest either optimization or using 5 residues instead.
